# Closing the Cultural Gap: An Intercultural Day Clinic Experience

**DOI:** 10.1192/j.eurpsy.2025.1661

**Published:** 2025-08-26

**Authors:** P. S. Sirin, L. Yükseltan-Hahn, S. D. Thakkar

**Affiliations:** 1 Zentrum für Seelische Gesundheit, Groß- Borstel, Asklepios Klinik Nord- Ochsenzoll, Hamburg, Germany

## Abstract

**Introduction:**

Globally, the number of international migrants has been rising, with Europe seeing a significantly higher increase compared to other regions. Migration has been frequently identified in the literature as a risk factor for various mental health issues (Schouler-Ocak et al. Indian J Psychiatry 2020; 62 242-6). Despite this, migrant populations often encounter significant challenges in accessing mental health care services, primarily due to language and communication barriers and cultural differences (Forray et al. BMC Public Health 2024; 24 1593). To improve mental health treatment for migrant populations, it is essential to not only provide cultural competence training for healthcare professionals and ensure access to professional interpreters but also to establish and maintain multicultural treatment teams (Machleidt W. Der Nervenarzt 2022; 73 1208-12). Addressing these needs, we have been operating a multicultural treatment team at our day clinic in Hamburg for the past year, providing care to Turkish-speaking migrants and native-born patients together.

**Objectives:**

To describe our efforts in developing a cross-cultural center and facilitating effective communication between migrant patients and native-born patients.

**Methods:**

A descriptive overview of our efforts to establish a day clinic model adapted to the cultural and linguistic needs of the migrant (Turkish) population in Germany with a brief review of the relevant literature.

**Results:**

At our day hospital, we provide care for patients with psychiatric disorders who do not require inpatient treatment but for whom outpatient care is insufficient. Our multicultural treatment team is composed of healthcare professionals whose native languages are German and/or Turkish.In our day hospital, patients with a migration background receive psychotherapy and medical consultations in their native language, ensuring they can access the treatment they need without language barriers. Additionally, we aim to improve cultural understanding through collaborative activities. This approach facilitates the development of cross-cultural communication among patients and healthcare professionals from different backgrounds, while also contributing to equal opportunities in psychiatric treatment. The program addresses linguistic, cultural, and religious communication difficulties, aiming to build and sustain meaningful relationships.

**Conclusions:**

The migrant population in Europe continues to grow each day, and mental health care services must adapt to this heterogeneous population and their diverse treatment needs. We advocate for the establishment of treatment centers where migrant populations and native-born patients are considered together, as such centers can play a role in bridging the intercultural communication gap.

**Disclosure of Interest:**

None Declared

